# Identification of Reassortant Mammalian Orthoreovirus Strains in European Hedgehogs (*Erinaceus europaeus*): Genomic Insights and Host Association

**DOI:** 10.3390/microorganisms13092047

**Published:** 2025-09-03

**Authors:** Tiziana Trogu, Maya Carrera, Clara Tolini, Ambra Nucci, Sabrina Canziani, Guido Grilli, Maria Cristina Rapi, Sara Manfredini, Silva Rubini, Davide Lelli, Valentina Carta, Cristina Bertasio, Enrica Sozzi, Antonio Lavazza, Ana Moreno

**Affiliations:** 1 Virology Department, Istituto Zooprofilattico Sperimentale della Lombardia e dell’Emilia Romagna—IZSLER, Via Bianchi, 9, 25124 Brescia, Italy; tiziana.trogu@izsler.it (T.T.); maya.carrera@izsler.it (M.C.); clara.tolini@izsler.it (C.T.); ambra.nucci@izsler.it (A.N.); sabrina.canziani@izsler.it (S.C.); davide.lelli@izsler.it (D.L.); enrica.sozzi@izsler.it (E.S.); alavazza0@gmail.com (A.L.); 2 Department of Veterinary Medicine and Animal Sciences, University of Milan, Via dell’Università 6, 26900 Lodi, Italy; guido.grilli@unimi.it (G.G.); maria.rapi@unimi.it (M.C.R.); sara.manfredini@unimi.it (S.M.); 3 Diagnostic Laboratory of Ferrara, Istituto Zooprofilattico Sperimentale della Lombardia e dell’Emilia Romagna—IZSLER, Via Modena, 483, Cassana, 44044 Ferrara, Italy; silva.rubini@izsler.it; 4 Genomic Analysis Laboratory, Istituto Zooprofilattico Sperimentale della Lombardia e dell’Emilia Romagna—IZSLER, Via Bianchi, 9, 25124 Brescia, Italy; valentina.carta@izsler.it (V.C.); cristina.bertasio@izsler.it (C.B.)

**Keywords:** mammalian orthoreovirus, hedgehogs, Italy, genomic characterization, reassortment

## Abstract

Thanks to its ethological and physiological characteristics, the hedgehog is a synanthropic species of particular importance for the maintenance and possible spread of pathogens, some of which are zoonotic. Among these, we can include the mammalian orthoreovirus (MRV), which is characterized by respiratory, gastrointestinal, and neurological symptoms in both animals and humans. MRV is characterized by a high capacity for genetic reassortment and intragenic rearrangement, and the ability to infect a wide range of mammals. This work aims to investigate the presence of MRVs and its genomic characterization in hedgehogs. During the two-year period from 2022 to 2023, the intestine and lungs were collected from 293 hedgehogs and subjected to real-time PCR to detect the L1 gene. Positive samples were subjected to a typing RT-PCR targeting a portion of the S1 gene and then to sequencing. A total of 38 hedgehogs tested positive by real-time PCR (*p* = 13%). Typing RT-PCR demonstrated the positivity of 25 samples for serotype 3. Four samples, representative of the main groups recognized during the phylogenetic analysis, underwent whole genome sequencing, revealing the presence of reassortment phenomena between strains related to bats, chamois, and human MRVs.

## 1. Introduction

The western hedgehog (*Erinaceus europaeus*, hereafter referred to as “hedgehog”) belongs to the order Eulipotyphla and possesses peculiar characteristics that make it particularly interesting from an ecopathological perspective. Its presence has been reported primarily in the western part of continental Europe, extending as far as Russia. It has also been observed on some Mediterranean islands, in the Azores, and has been introduced in New Zealand [1].

Although widespread, the hedgehog is classified as “Near Threatened” by the International Union for Conservation of Nature (IUCN) [2] due to its demographic decline in recent decades in many European countries [3,4]. An interesting aspect of hedgehog population dynamics is that this generalized decline has been mainly recorded in rural areas, accompanied by a progressive displacement of individuals to more urbanized environments [5,6,7]. These dynamics result from various changes in recent years, such as alterations in agricultural land use [8], and habitat fragmentation, as well as the presence, and in some cases, expansion of potential predators [7,9], and the increasing availability of food for domestic animals and potential refuges [5]. All these factors have contributed to the progressive adaptation of this species to urban and peri-urban areas. The hedgehog’s versatility in finding new ecological niches in urban settings leads to a predominant overlap with humans and their pets. It may also act as a bridge between wild species inhabitation transitional zones and those living in more rural areas that still exhibit a high degree of urbanization (e.g., small carnivores or rodents).

From a public health perspective, increased opportunities for contact between humans and urban wildlife could facilitate the interspecific transmission of various pathogens, potentially leading to new outbreaks of zoonotic diseases [10]. Several reviews have shown that hedgehogs can harbor numerous pathogens, some of which are potentially zoonotic [11,12]. Indeed, the ethological and biological characteristics of this species make it a possible reservoir, or even amplifier, of specific microorganisms. For example, following the SARS-CoV-2 pandemic, numerous studies have been carried out to detect and characterize coronaviruses in wild animals. It has been suggested that hedgehogs may also act as reservoirs for such viruses [13,14].

Among other potentially zoonotic viruses that can be transmitted by wild and domestic animals through orofecal and respiratory routes are the mammalian orthoreoviruses (MRVs) [15]. Although the genus Orthoreovirus comprises different species [16], MRVs represent the first Orthoreovirus that was isolated from humans in 1950 [17]. MRVs include three major serotypes, which are characterized by different abilities of elicited antibodies to neutralize viral infectivity and inhibit hemagglutination [17]. Symptoms associated with MRV infections are primarily respiratory, gastrointestinal, and neurological, and occur in both animals [18,19] and humans [15,16,17,18,19,20,21].

The strength of this virus lies in its susceptibility to genetic reassortment and intragenic rearrangement phenomena [22,23,24], as well as its ability to infect a wide range of mammals [25,26,27,28,29,30], including humans [15]. Numerous reports have documented MRVs in wildlife globally. In Italy, several studies have demonstrated the presence of MRVs in various wild species, including wild ungulates [31,32] and synanthropic species, such as bats [24,25] and mice [33]. The information gathered on the presence of this virus in wildlife in northern Italy suggests that other species may also be involved in its epidemiological cycle, especially considering its natural tendency to undergo mutations and genomic reassortments. For this reason, the present study focused on searching for MRVs in hedgehogs, a species known to be synanthropic and recently subject to increased awareness and, consequently, human manipulation. Currently, there is no information on the presence, frequency, or distribution of these viruses in hedgehog populations. However, this species could potentially act as a reservoir or play an epidemiological role in the transmission of the virus.

## 2. Materials and Methods

### 2.1. Sampling

During the two-year period from 2022 to 2023, a total of 293 hedgehogs that died during hospitalization at wildlife rehabilitation centers (WRCs) were submitted to the Istituto Zooprofilattico Sperimentale della Lombardia e dell’Emilia Romagna (IZSLER) for necropsy and determination of the cause of death, with a specific focus on diseases and pathogens included in regional monitoring plans [34]. The hedgehogs originated from four different rehabilitation centers operating at the provincial level, with three located in Lombardy and one in the Emilia Romagna region.

### 2.2. MRV Diagnostic Approaches and Sequence Strategies

To investigate the presence of viruses belonging to the genus Mammalian Orthoreovirus, intestine and lungs were collected from each carcass and homogenized (1:10 dilution *w*/*v*) in phosphate-buffered saline containing 1% penicillin and streptomycin and 10% glycerol. After centrifugation at 3750 rpm for 15 min, 250 µL of supernatant was used for viral genome extraction using the QIAsymphonyTM SP Instrument (Qiagen, Hilden, Germany) according to the manufacturer’s instructions. Negative and positive controls were included in the extraction.

Viral nucleic acid from all the samples was subjected to an additional denaturing step of 95 °C for 5 min, and subsequently to screening real-time RT-PCR, targeting the L1 segment [35], using SuperScript™ (Thermo Fisher Scientific, Waltham, MA, USA). The following cycler conditions were used: 45 °C for 10 min, 95 °C for 10 min, and 45 amplification cycles (95 °C for 15 s and 60 °C for 45 s).

Samples that tested positive in the initial screening were first subjected to virological examination on VERO and MARC-145 cells, both derived from the kidney of the African green monkey. The homogenized organs were centrifuged to allow sedimentation of corpuscles; the supernatant was filtered through a 45 µm filter. Three wells of monolayer cell cultures for each cell line, with a surface area of 1.93 cm^2^, were inoculated with 150, 100 and 50 µL of the sample, respectively. The cell monolayers were observed until cytopathic effects appeared or for up to three consecutive passages. Positive samples were then confirmed using a previously described real-time RT-PCR. To type the detected viruses, the samples were subjected to RT-PCR that targeted a portion of the S1 segment, which identifies the three major MRV serotypes [28] and encodes the σ1 protein involved in viral attachment [36]. The amplicon sizes of 505 bp, 394 bp, and 326 bp correspond to serotypes 1, 2 and 3, respectively. Only the samples that were positive for serotype 3 were further tested using an additional PCR that was capable of amplifying the entire S1 segment of MRV3 [28]. Table 1 reports the sequence of primers and probes used for each PCR assay.

PCR products were processed, sequenced, and purified with Exonuclease I and Thermosens Phosphatase alkaline (FastAp), the Big dye^®^ terminator ready reaction v1.1 kit, and the BigDye^®^ Xterminator Purification kit (Thermo Fisher Scientific, Waltham, MA, USA), respectively. The run was performed on a SeqStudio platform (Thermo Fisher Scientific, Waltham, MA, USA), and the quality of electropherograms was assessed for both forward and reverse sequences using Sequencing Analysis v5.4 software. Sequencing data (.ab1 format file) were analyzed using the Lasergene software SeqMan module (DNAStar, Madison, MI, USA, versions from 15 to 17).

WGS (whole genome sequencing) was performed on the extracted samples: after RNA quantification with the QuantiFluor^®^ RNA System on a Quantus™ Fluorometer (Promega, Fitchburg, MA, USA), libraries were prepared using Illumina Stranded Total RNA Prep with the Ribo-Zero Plus kit (Illumina, San Diego, CA, USA) and were sequenced on an Illumina MiniSeq platform. NGS analysis was performed on the raw reads, and a contemplated quality check was performed with FastQC (v0.12.1; https://github.com/s-andrews/FastQC, accessed on 11 November 2024). Furthermore, de novo assembly was performed with Unicycler (v0.5.1) using the default parameters and alignment against reference genome segments from NC077837 to NC077846 using Geneious prime (v2024.0.5) with the default setting. The consensus sequences obtained from whole genome sequencing (WGS) were analyzed using the NCBI BLASTn tool (https://blast.ncbi.nlm.nih.gov/, accessed on 11 November 2024).

Partial S1 gene sequences were obtained by Sanger sequencing and used for phylogenetic analysis. A dataset comprising Italian hedgehog MRV3 sequences and reference S1 gene sequences from human and animal MRV3 strains from Europe, Asia, and the Americas was compiled from GenBank (https://www.ncbi.nlm.nih.gov/genbank/, accessed on 11 November 2024). Sequences were aligned using ClustalW implemented in MEGA X [37]. Phylogenetic trees were constructed using the maximum likelihood (ML) method with IQ-TREE (http://iqtree.cibiv.univie.ac.at/, accessed on 11 November 2024) [38]. Tree robustness was assessed by bootstrap analysis with 1000 replicates. The trees were visualized using FigTree v1.4.4 (http://tree.bio.ed.ac.uk/software/figtree/, accessed on 11 November 2024). All gene segments of the four fully sequenced Italian MRV3 samples were analyzed individually using the NCBI BLASTn tool to assess their nucleotide similarity with sequences in the public database.

## 3. Results

From the necropsy results, most of the hedgehogs (approximately 44%) did not show lesions that were referable to infectious diseases. In some cases, the animals were in an advanced state of decomposition or exhibited significant cadaveric alterations. About 13% of the hedgehogs showed splenomegaly, which was sometimes accompanied by pneumonia or enteritis. A low percentage of animals (4%) had ectoparasites, such as ticks and fleas. For approximately 20% of the animals, necropsy investigation results were not available.

The real-time RT-PCR results revealed the presence of MRV in 38 hedgehogs (*p* = 13%, CI 95%:10–17), especially in four animals collected in 2022 (*p* = 3.5%, CI 95%:1.3–8.6) and 34 in 2023 (19%, CI 95%:13–25). Table 2 summarizes the positive and negative samples from the real-time RT-PCR screening.

**Figure 1:**
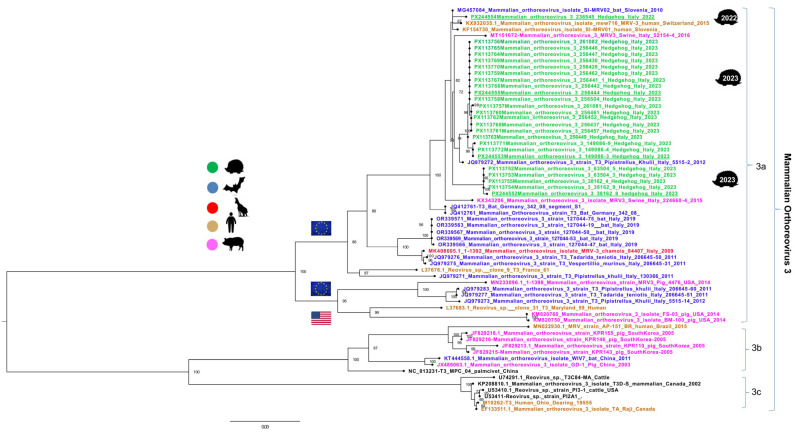
Maximum likelihood phylogenetic tree based on partial S1 gene sequences of mammalian orthoreovirus type 3 (MRV3). Sequences are color-coded according to host origin. Sequences derived from hedgehogs in this study are highlighted in green, and the four fully sequenced samples are indicated with underlining. The ML tree was inferred using the IQ-TREE web server with 1000 bootstrap replicates. Bootstrap values ≥ 70 are shown at the corresponding nodes. The host species and year of isolation are indicated in the name of each sequence. The accession numbers of Italian sequences are listed next to the name. The fully sequenced Italian MRV3 strains are underlined.

In 19 out of the 38 samples, a cytopathic effect was observed in the cell monolayer, and the viable virus was confirmed as MRV by RT-PCR screening in all cases. Additionally, all 38 PCR-positive samples were subjected to a typing RT-PCR, which identified 25 samples as serotype 3. The remaining 13 samples could not be typed. This typing PCR [28] has previously been shown to be effective for typing serotype 3 viruses, but is less sensitive for other serotypes. Consequently, 25 full S1 sequences were obtained. The phylogenetic analysis revealed a close relationship with MRV serotype 3 sequences isolated in 2012 in Italy from bats of the genus *Pipistrellus khulii* (Figure 1). Despite clustering together, three slightly distinct subgroups emerged from the sequences’ comparison. Therefore, four representative samples from the main groups recognized were selected for whole genome sequencing.

**Figure 2:**
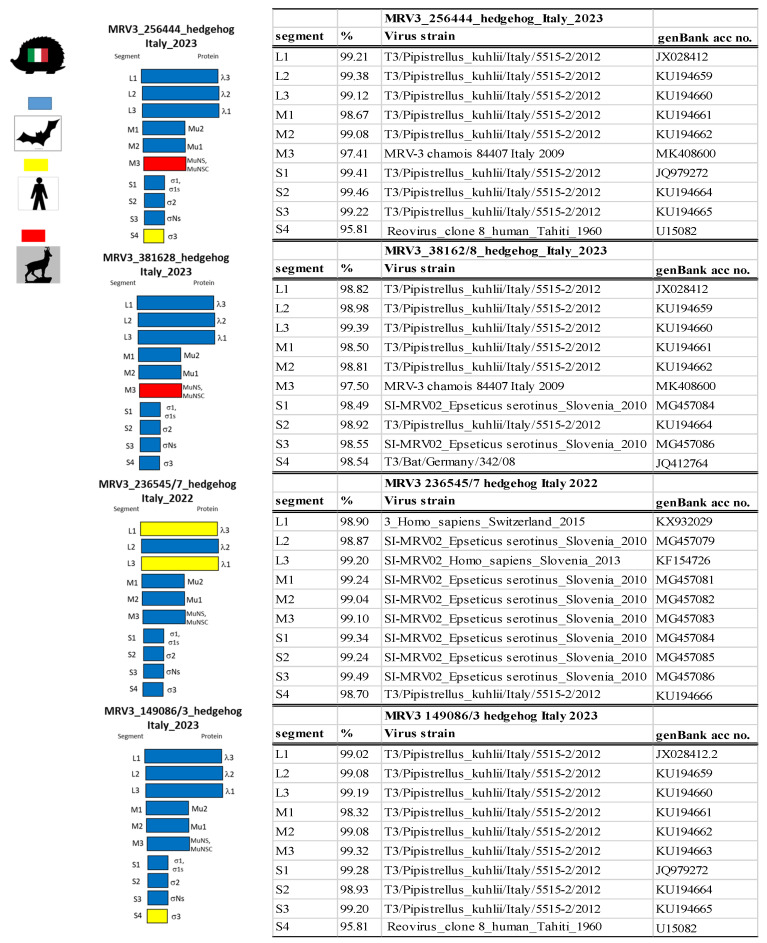
Genome segment constellations of fully sequenced MRV3 strains detected in Italian hedgehogs and nucleotide identity (%) of each segment to the closest matching sequences in GenBank. Each segment (L1–L3, M1–M3, and S1–S4) is represented separately. Colors indicate the species origin of the most closely related strain for each segment (red for sequences similar to those isolated from chamois, blue from bats, and yellow from humans), highlighting possible reassortment events and interspecies transmission. The accession numbers of Italian sequences are listed below in the order of the gene segments L1, L2, L3, M1, M2, M3, M4, S1, S2, S3, and S4: MRV3_256444: PX244571; PX244567; PX244563; PX244587; PX244591; PX244559; PX244555; PX244583; PX244579; PX244575. MRV3_38162/8: PX244568; PX244564; PX244560; PX244584; PX244588; PX244556; PX244552; PX244580; PX244576; PX244572. MRV3_236545/7: PX244570; PX244566; PX244562; PX244586; PX244590; PX244558; PX244554; PX244582; PX244578; PX244574. MRV3_149086/3: PX244569; PX244565; PX244561; PX244585; PX244589; PX244557; PX244553; PX244581; PX244577; and PX244573.

Figure 2 summarizes the results of the genetic analyses, while phylogenetic trees for each gene segment are presented in the Supplementary Files. Briefly:

- The first MRV sequence (MRV3_256444), identified in the Como province in 2023, had segment M3 closely related to a sequence from Italian chamois (MRV-3 chamois 84407 Italy 2009), while segment S4 showed 95.81% identity with a human strain from Tahiti. The remaining segments were related to a bat strain isolated from *Pipistrellus kuhlii* (T3/Pipistrellus_kuhlii/Italy/5515-2/2012), which was detected in Modena province (Emilia Romagna region) in 2012 [25].
- The second sample (MRV3_38162/8), collected in Varese province, displayed greater variability in segment composition. Segment M3 was again related to the Italian chamois strain, and most of the remaining segments were similar to the *P. kuhlii* strain. However, segments S1 and S3 were related to a strain from *Eptesicus serotinus* in Slovenia, while segment S4 was related to a bat-derived strain from Germany.
- The third MRV sequence (MRV3_236545/7), from Brescia province, had segments L1 and L3 related to human strains from Switzerland and Slovenia, respectively. Interestingly, aside from the final segment—similar to T3/Pipistrellus_kuhlii/Italy/5515-2/2012—all other segments were related to a strain detected in *Eptesicus serotinus* from Slovenia.
- The fourth MRV sequence (MRV3_149086/3), also from Brescia province, was predominantly related to T3/Pipistrellus_kuhlii/Italy/5515-2/2012, with only segment S4 showing a relationship to the human strain from Tahiti.

## 4. Discussion

This study reports the detection of mammalian orthoreovirus in the European hedgehog, with an overall positivity rate of 13%. Typing RT-PCR identified MRV serotype 3 in 25 samples, while the remaining 13 samples could not be typed. Considering that virus isolation was also unsuccessful in most of these cases, the failure of typing likely resulted from virus degradation, which prevented accurate identification. To our knowledge, this represents the first identification of MRVs in this species. Due to the novelty of this finding, it is difficult to define the epidemiological context of MRV infection in hedgehogs. This is further limited by the absence of additional data on the sampled animals, such as sex, age, and morphometric measurements. The only available information was the municipality of provenance, which was not georeferenced.

Furthermore, it cannot be ruled out that animals hospitalized in rescue centers may have contracted the infection through contact with other hedgehogs or with infected species present within the facility. This possibility further complicates the interpretation of the epidemiological framework. Currently, little is known about the clinical or pathological implications of MRV infection in hedgehogs. From an anatomopathological perspective, attributing specific alterations to MRV infection is difficult, as only one positive animal showed splenomegaly. The remaining hedgehogs either presented no macroscopic lesions or were too poorly preserved for reliable lesion assessment, making it difficult, if not impossible, to identify a potential cause of death.

MRVs are geographically widespread viruses with a broad host range. However, their pathogenic role in hedgehogs—and in many wild species—remains controversial. While some studies associate MRVs with symptomatic or asymptomatic infections in humans [15] and animals [39], experimental infections in mice have demonstrated clinical outcomes that range from respiratory distress and weight loss to death, depending on the viral load [40]. In humans, recent evidence links MRVs to severe clinical presentations, including diarrhea, respiratory tract infections, and encephalitis [20,41].

The broad host range of MRVs is supported by their environmental resilience. These viruses can persist on surfaces, in food, and in water [42,43], enabling indirect transmission in addition to direct contact. Together with their capacity to cross species barriers, this increases the number of susceptible hosts and the likelihood of reassortment events. Reassortment is a key evolutionary mechanism in MRVs, which possesses a segmented genome of ten double-stranded RNA segments. Coinfection of a host cell by different strains can result in genomic segment exchange, generating novel viral genotypes. Recombination, a common process in RNA viruses, further enables genetic modifications that may allow the virus to resist and overcome selective pressures and adapt to new hosts and environments [44]. Conversely, such virus mutations could also lead to unpredictable biological and evolutionary consequences, including increased virulence or zoonotic potential. Several cases of MRV reassortants have been reported in different species [23,26,29,32,45]; notably, this phenomenon is particularly important in bats due to their biological characteristics and their epidemiological role in the transmission and spreading of viral agents [24,46,47]. In fact, reassortant MRVs have been observed in a specific bat genus (*Rhinolophus*) [24,46], leading to the hypothesis that host species may actively contribute to recombination processes [24].

In this study, the genomic characterization of four fully sequenced MRV strains from hedgehogs revealed distinct segment profiles. Most segments, however, exhibited the highest nucleotide identity with bat-derived MRV sequences. This strongly suggests that this virus is well adapted to chiroptera, facilitating its spread across bat populations. This would explain the similarity with MRV sequences from bats sampled in areas geographically distant from the sampled hedgehogs.

The strong phylogenetic link between hedgehog and bat MRVs also reflects the ecological and biological similarities as well as the phylogenetic proximity between the two species [48]. Both hedgehogs and bats are insectivorous, nocturnal, and capable of hibernation—suggesting an evolutionary link that may influence their role as viral reservoirs. The hedgehog’s highly synanthropic behavior, often living in urban and peri-urban areas, bringing it into close contact with humans and domestic and wild animals, makes it an ideal candidate not only as a reservoir but also as a bridge species for MRV transmission. This is further supported by other genomic segments that show homology with MRVs from humans and chamois, indicating past reassortment events. Notably, the Italian bat-derived strain T3/Pipistrellus_kuhlii/Italy/5515-2/2012 has been shown to reassort with strains from pigs, minks, and humans [24], acting as a donor of segments such as S1, M1, and S4—a pattern that is also observed in the hedgehog strains in this study. Furthermore, MRV-3 chamois/84407/Italy/2009 has contributed the M3 segment to other reassortant viruses infecting both humans and animals [32].

The phylogenetic relationships observed between hedgehog MRVs and sequences from diverse hosts and distant geographical areas suggest a slow but persistent viral evolution, shaped by the long-term circulation of MRVs and the movement of animals and humans. The genetic similarity with a strain from Alpine chamois may reflect territorial proximity, as both the chamois and the MRV-positive hedgehogs were sampled in northwestern Italy—albeit in different ecosystems. Indeed, it is plausible that other species with wider ranges could have contributed to viral spread.

In comparison to human-derived strains, it is possible that certain viral lineages were introduced into Italy in the past and maintained in under-sampled natural reservoirs, such as small mammals, preserving genomic segments over time. This highlights the need for broader viral surveillance efforts in wild animal populations to better understand the diversity, evolution, and geographic distribution of MRV.

The continued presence of MRVs in wild populations, along with their genomic plasticity through reassortment and potential recombination, underscores the dynamic nature of these viruses. MRVs are capable of adapting to new hosts and environments via genetic modifications that promote survival under selective pressures. While such changes can enhance viral persistence, they may also unpredictably increase virulence or the zoonotic potential.

## 5. Conclusions

The detection of MRVs in European hedgehogs is significant given their zoonotic potential, capacity for interspecies transmission, and extreme genetic plasticity. The presence of the virus in a synanthropic species raises concerns about possible spillover events, especially considering the high frequency with which hedgehogs are handled in wildlife rehabilitation centers. This study highlights the complexity of MRV infections in wildlife and underscores the need for ongoing surveillance and genomic analysis to monitor the emergence and evolution of new MRV strains with potential public health relevance.

## Figures and Tables

**Table 1:** Primers and probes used for molecular investigations.

Target [Ref.]	Primer	Sequence 5′ to 3′	MRV Specificity	Amplicon Size (bp)
L1 [35]	BatReoF	CACCATGTCAAGCTGCTCCC	All types	
	BatReoR	ACCGCCATGTATGTCCTCCAG		
	BatReoProbe	FAM-CCCAGTCGCGGTCATTACCACTCCG-BBQ		
S1 [28]	S1-R1F	GGAGCTCGACACAGCAAATA	Type 1	505
	S1-R1R	GATGATTGACCCCTTGTGC		
S1 [28]	S1-R2F	CTCCCGTCACGGTTAATTTG	Type 2	394
	S1-R2R	GATGAGTCGCCACTGTGC		
S1 [28]	S1-R3F	TGGGACAACTTGAGACAGGA	Type 3	326
	S1-R3R	CTGAAGTCCACCRTTTTGWA		
S1 [36]	ENT-S1-R3F	GCTATTGGTCGGATGGAT	Type 3	1416
	ENT-S1-R3R	GATGAAATGCCCCAGTGC		

**Table 2:** Hedgehogs collected in the provinces of Lombardy and Emilia Romagna, the two regions under the competence of the IZSLER, and results from the RT-PCR screening.

Province	Number of Hedgehogs				
	2022		2023		Total
	Neg	Pos	Neg	Pos	
Lombardy					
Bergamo	23	2	43	2	70
Brescia	29	2	7	3	41
Como			1	2	3
Cremona	2		1	4	7
Lecco	1		1		2
Lodi			3	5	8
Monza Brianza			1	1	2
Milan	2		20	11	33
Varese			1	5	6
Emilia Romagna					
Ferrara	53		65	1	119
Not defined			2		2
Total	110	4	145	34	293

## Data Availability

The original contributions presented in this study are included in the article/Supplementary Material. Further inquiries can be directed to the corresponding author.

## Supplementary Materials

The following supporting information can be downloaded at: https://www.mdpi.com/article/10.3390/microorganisms13092047/s1. Maximum likelihood phylogenetic trees constructed for each complete gene segment (L1, L2, L3, M1, M2, M3, S1, S2, S3, and S4). Reference MRV sequences were downloaded from GenBank, and the four fully sequenced Italian samples are included. Sequence names indicate the host species and year of isolation. Trees were generated using IQ-TREE software with 1000 bootstrap replicates. Italian sequences are highlighted in yellow. Figure S1: MRV gene L1 tree; Figure S2: MRV gene L2 tree; Figure S3: MRV gene L3 tree; Figure S4: MRV gene M1 tree; Figure S5: MRV gene M2 tree; Figure S6: MRV gene M3 tree; Figure S7: MRV gene S1 tree; Figure S8: MRV gene S2 tree; Figure S9: MRV gene S3 tree; Figure S10: MRV gene S4 tree.
