# Supplementary figures and images for: Identification of Reassortant Mammalian Orthoreovirus Strains in European Hedgehogs (Erinaceus europaeus): Genomic Insights and Host Association
Source: Microorganisms. 2025 Sep 3;13(9):2047. doi: 10.3390/microorganisms13092047 (PMC12473063; doi:10.3390/microorganisms13092047)

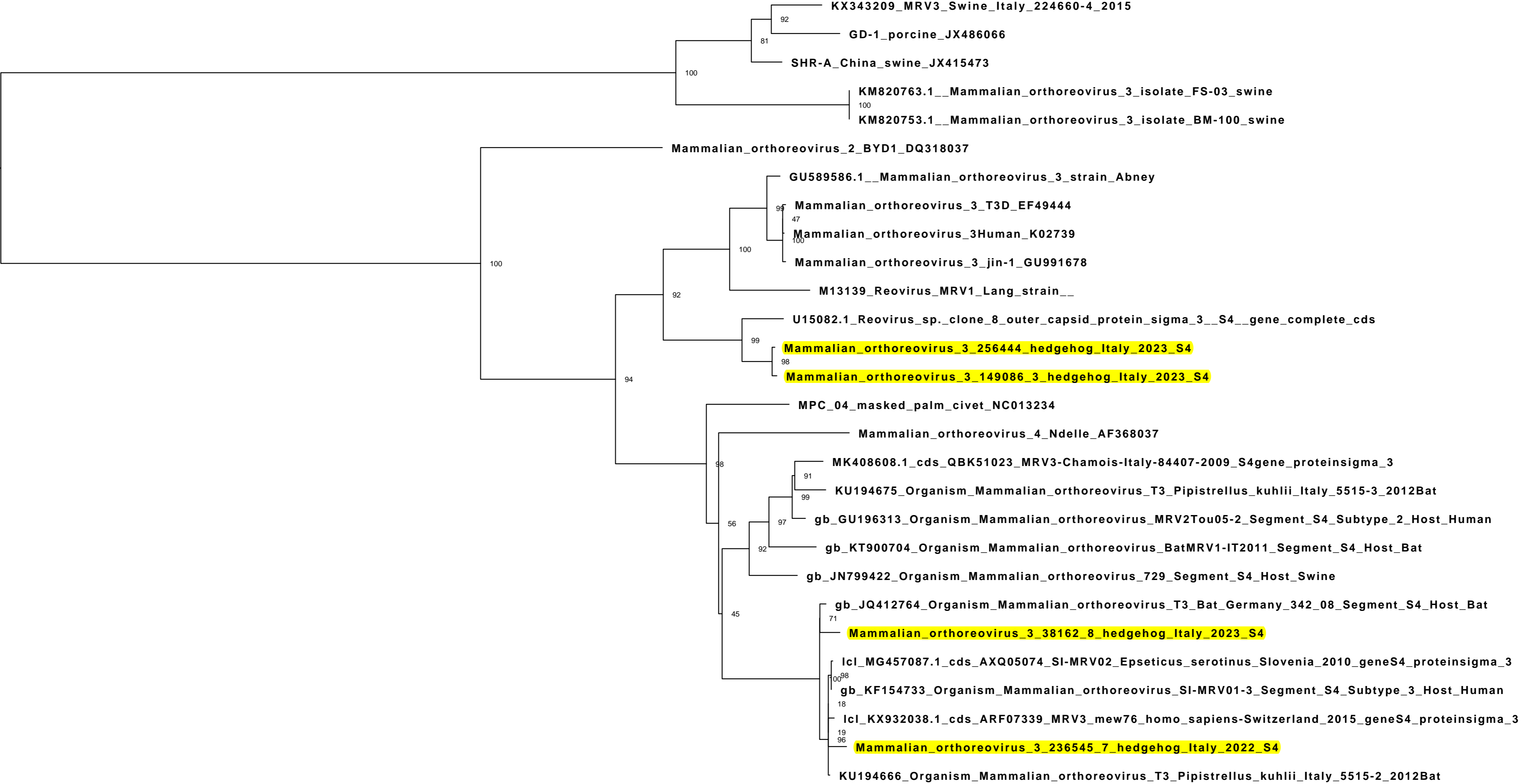

Supplement: Supplementary file 1 [file microorganisms-13-02047-s001.zip › Figure S10_MRV-S4tree.pdf]

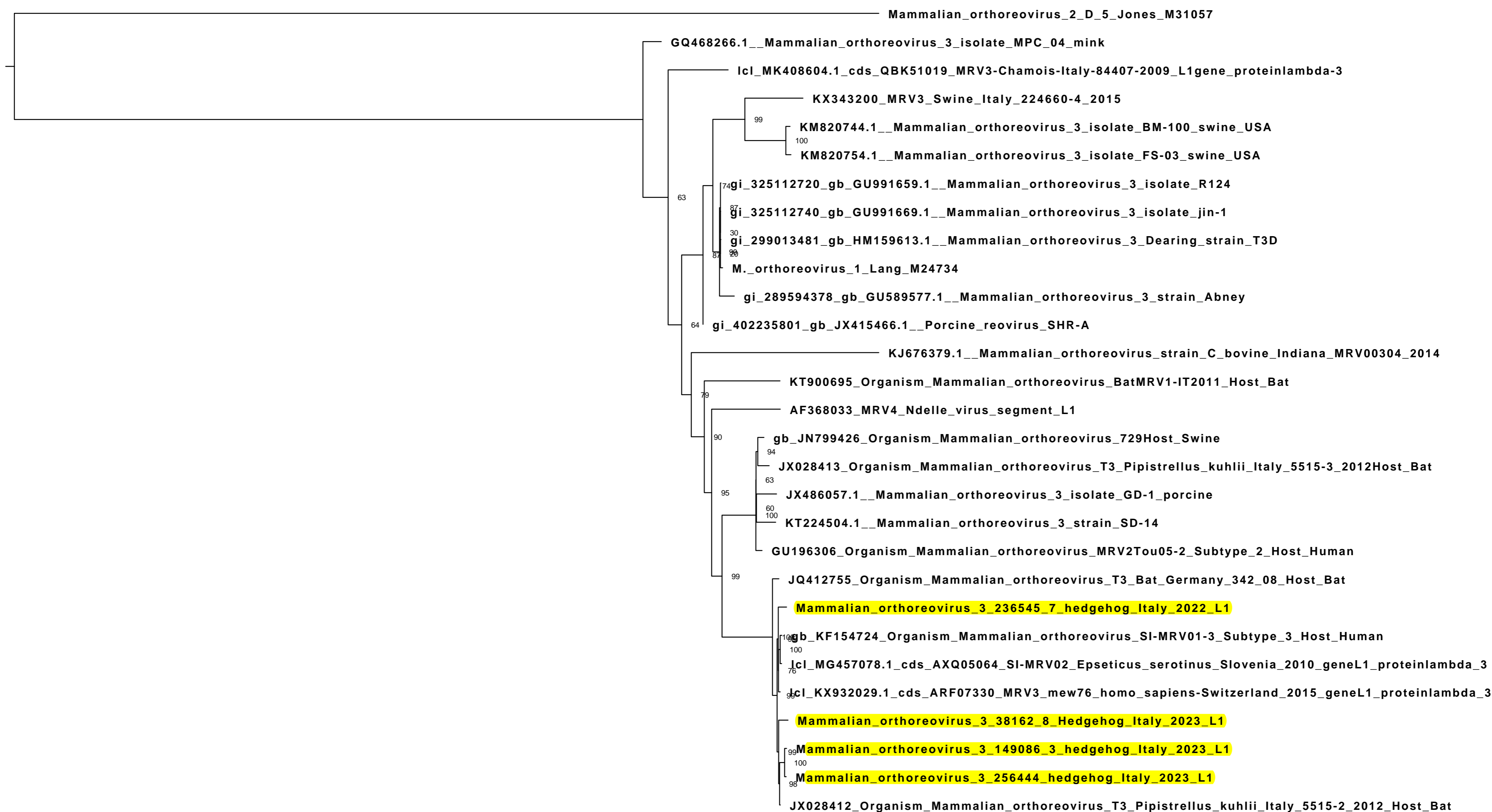

Supplement: Supplementary file 1 [file microorganisms-13-02047-s001.zip › Figure S1_MRV_L1-tree.pdf]

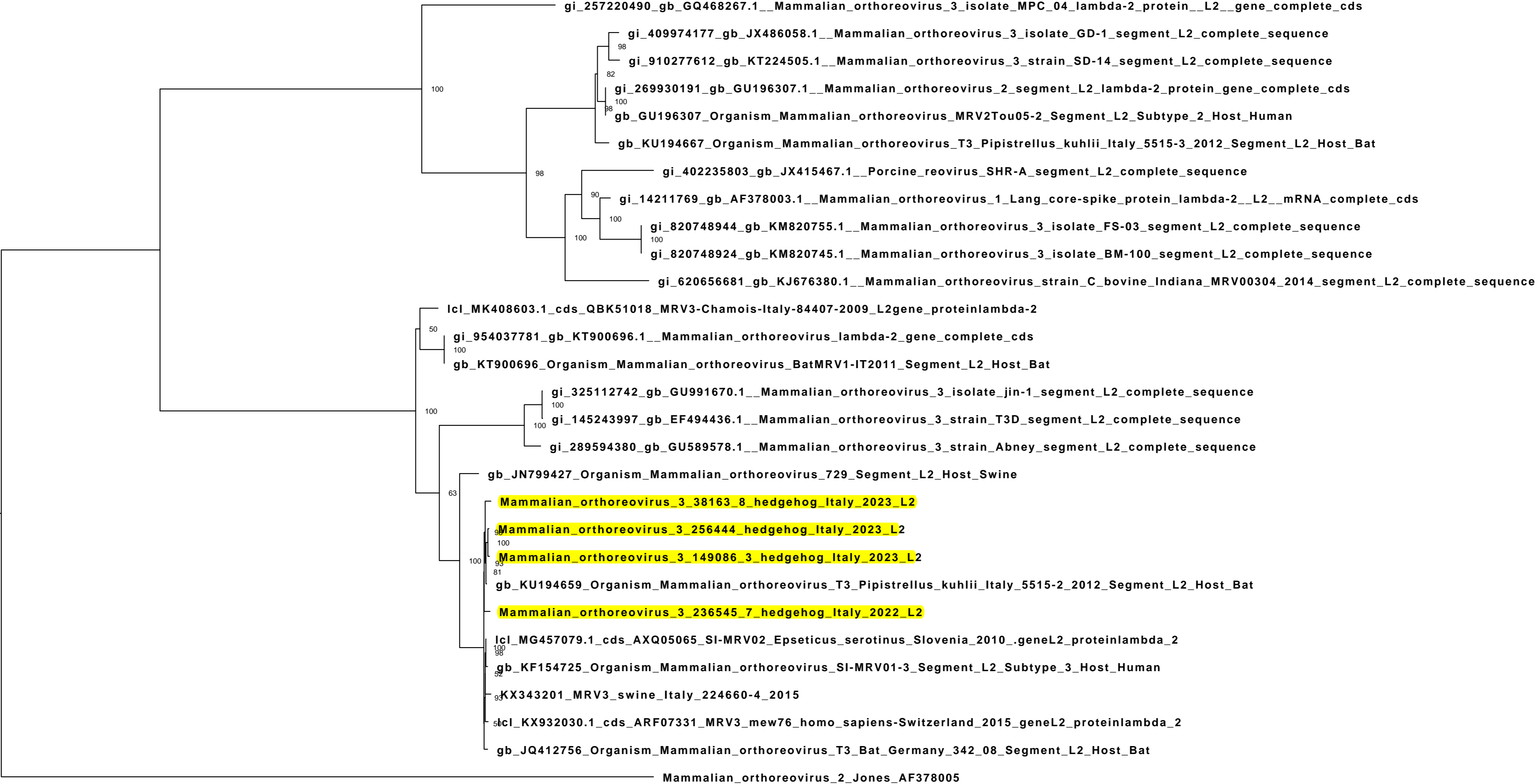

Supplement: Supplementary file 1 [file microorganisms-13-02047-s001.zip › Figure S2_MRV_L2-tree.pdf]

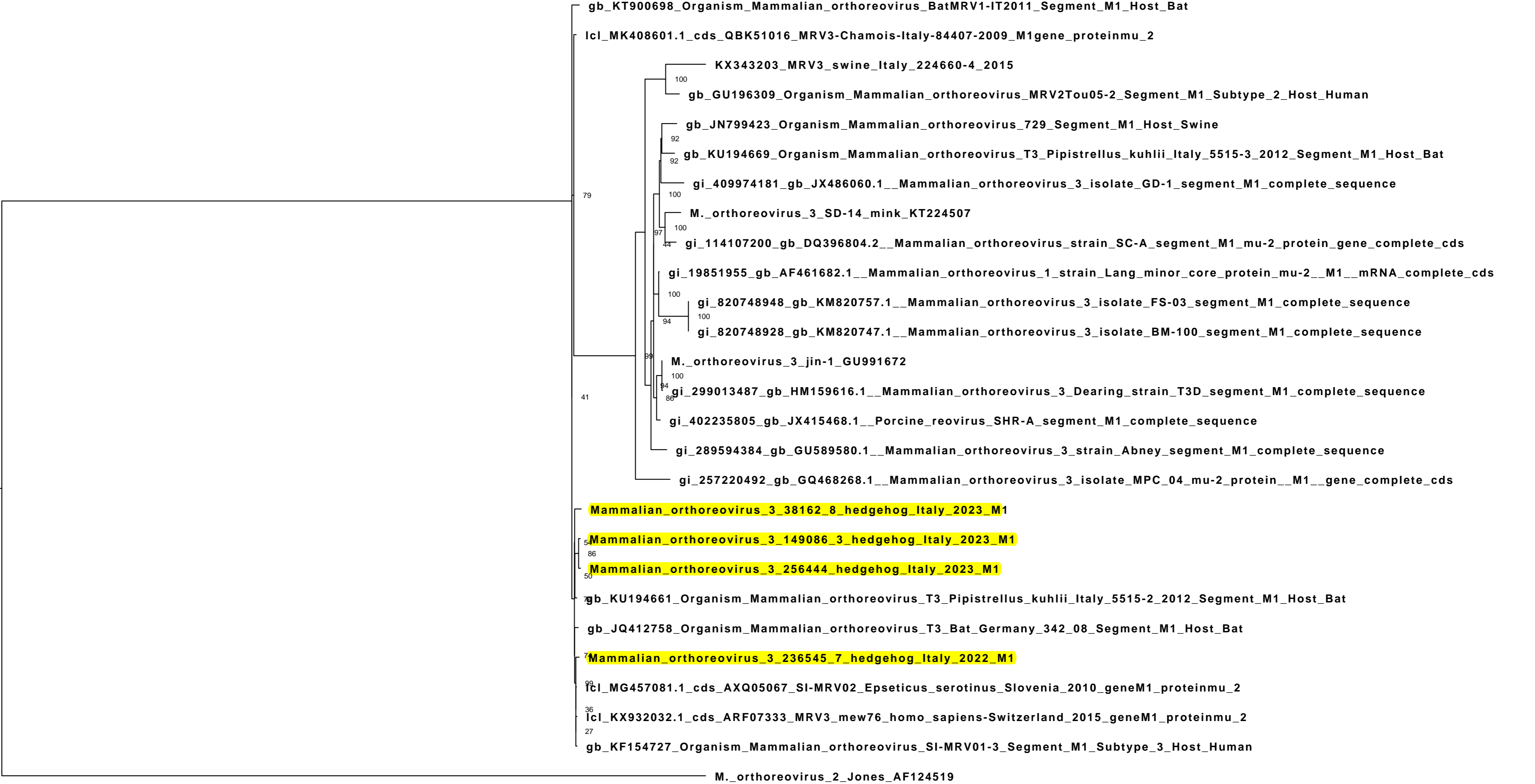

Supplement: Supplementary file 1 [file microorganisms-13-02047-s001.zip › Figure S4_MRV-M1tree.pdf]

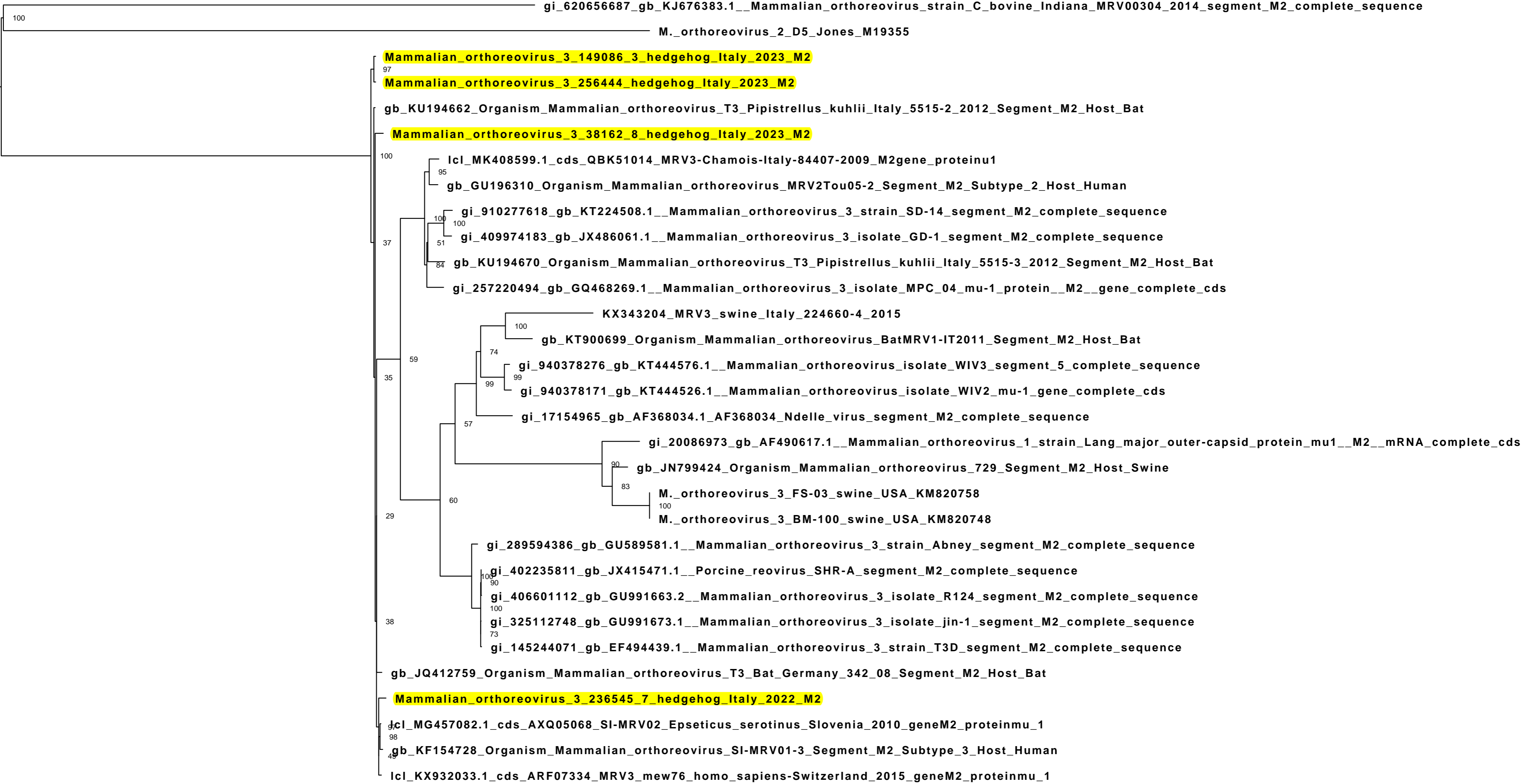

Supplement: Supplementary file 1 [file microorganisms-13-02047-s001.zip › Figure S5_MRV-M2tree.pdf]

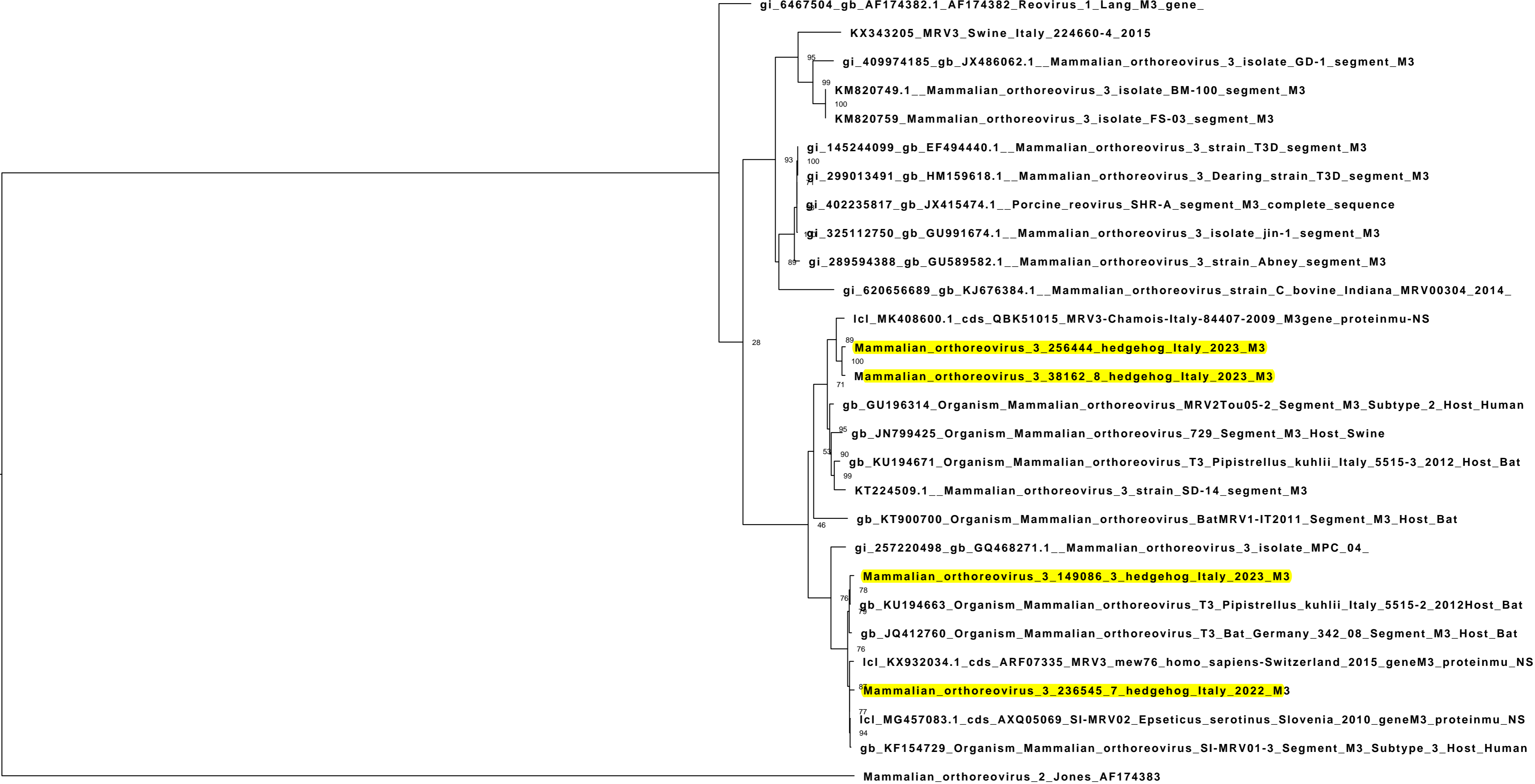

Supplement: Supplementary file 1 [file microorganisms-13-02047-s001.zip › Figure S6_MRV-M3tree.pdf]

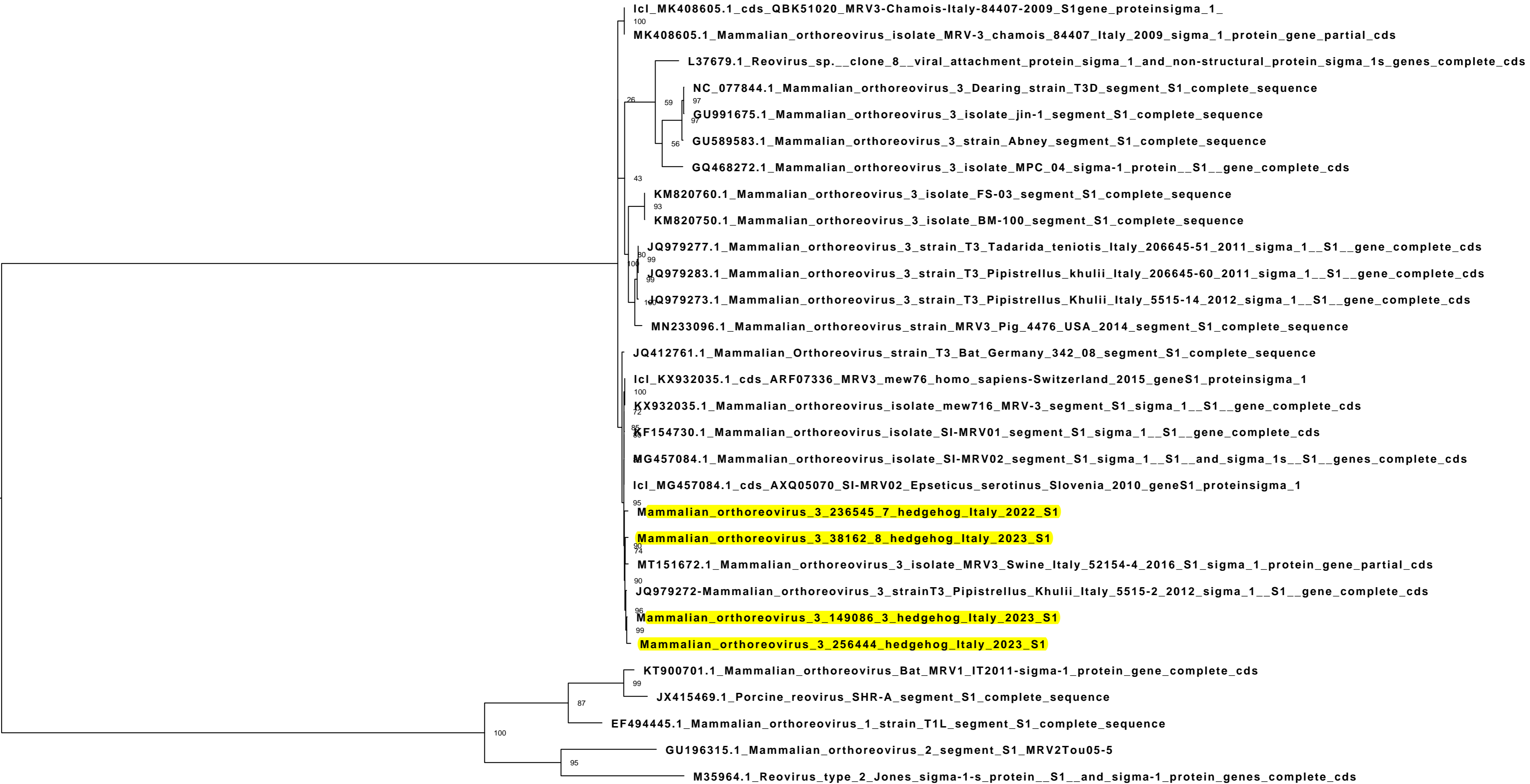

Supplement: Supplementary file 1 [file microorganisms-13-02047-s001.zip › Figure S7_MRV-S1tree.pdf]

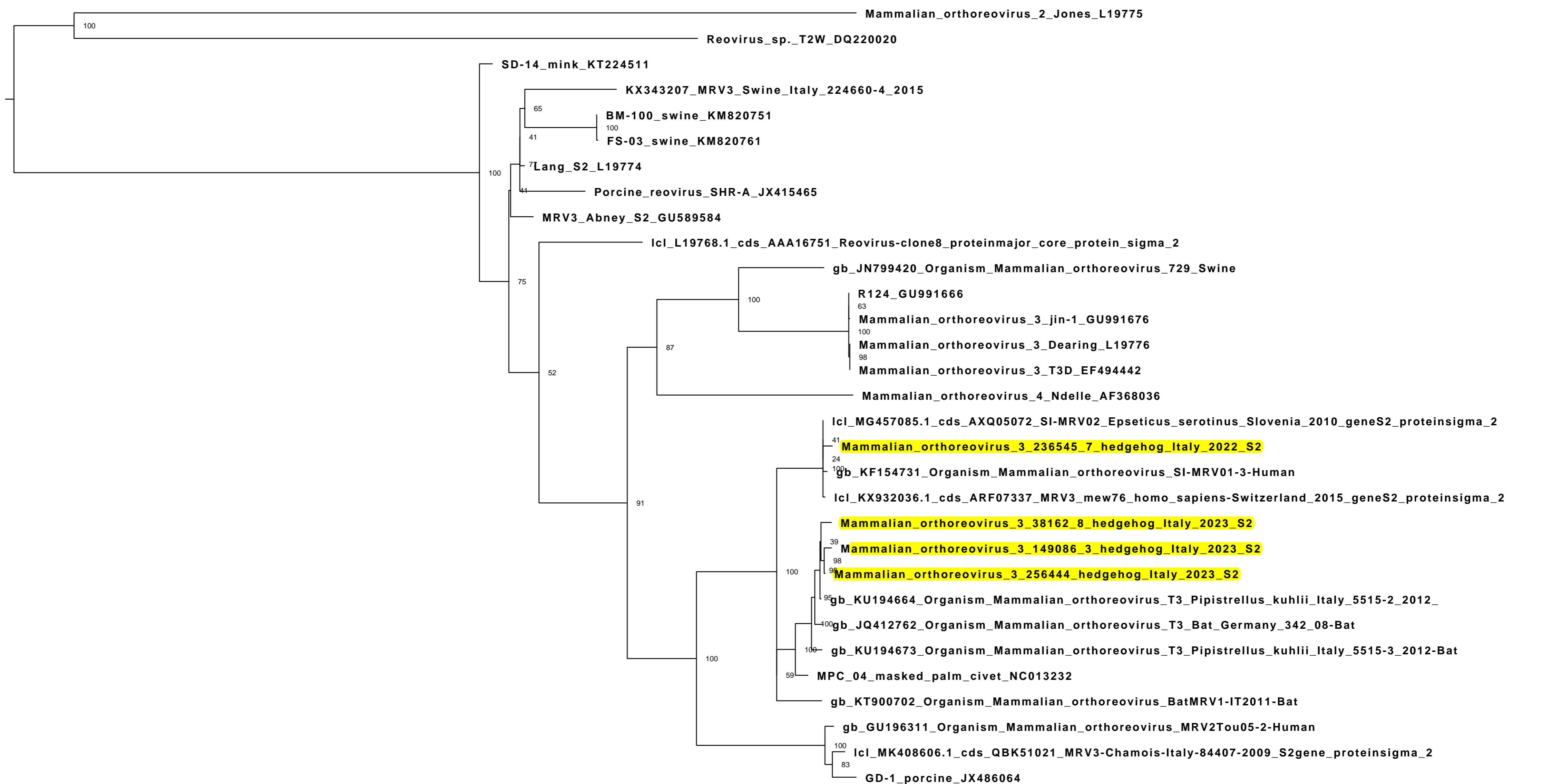

Supplement: Supplementary file 1 [file microorganisms-13-02047-s001.zip › Figure S8_MRV-S2tree.pdf]

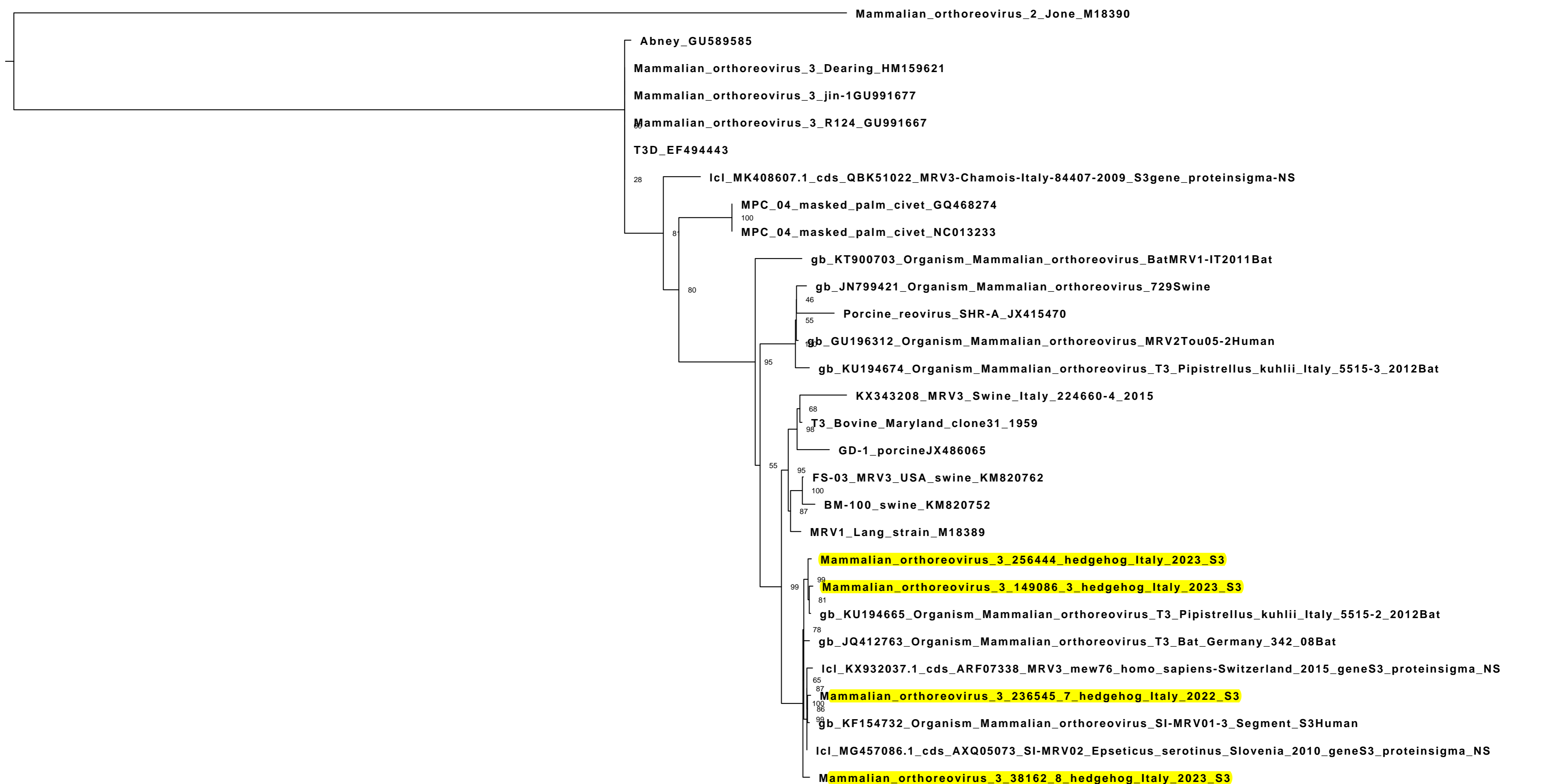

Supplement: Supplementary file 1 [file microorganisms-13-02047-s001.zip › Figure S9_MRV-S3tree.pdf]
