# Supplementary material for: Identification of Reassortant Mammalian Orthoreovirus Strains in European Hedgehogs (Erinaceus europaeus): Genomic Insights and Host Association
Source: Microorganisms. 2025 Sep 3;13(9):2047. doi: 10.3390/microorganisms13092047 (PMC12473063; doi:10.3390/microorganisms13092047)
Supplement: Supplementary file 1 [file Figure S3_MRV-L3-tree.pdf › Figure S3_MRV-L3-tree.pdf]

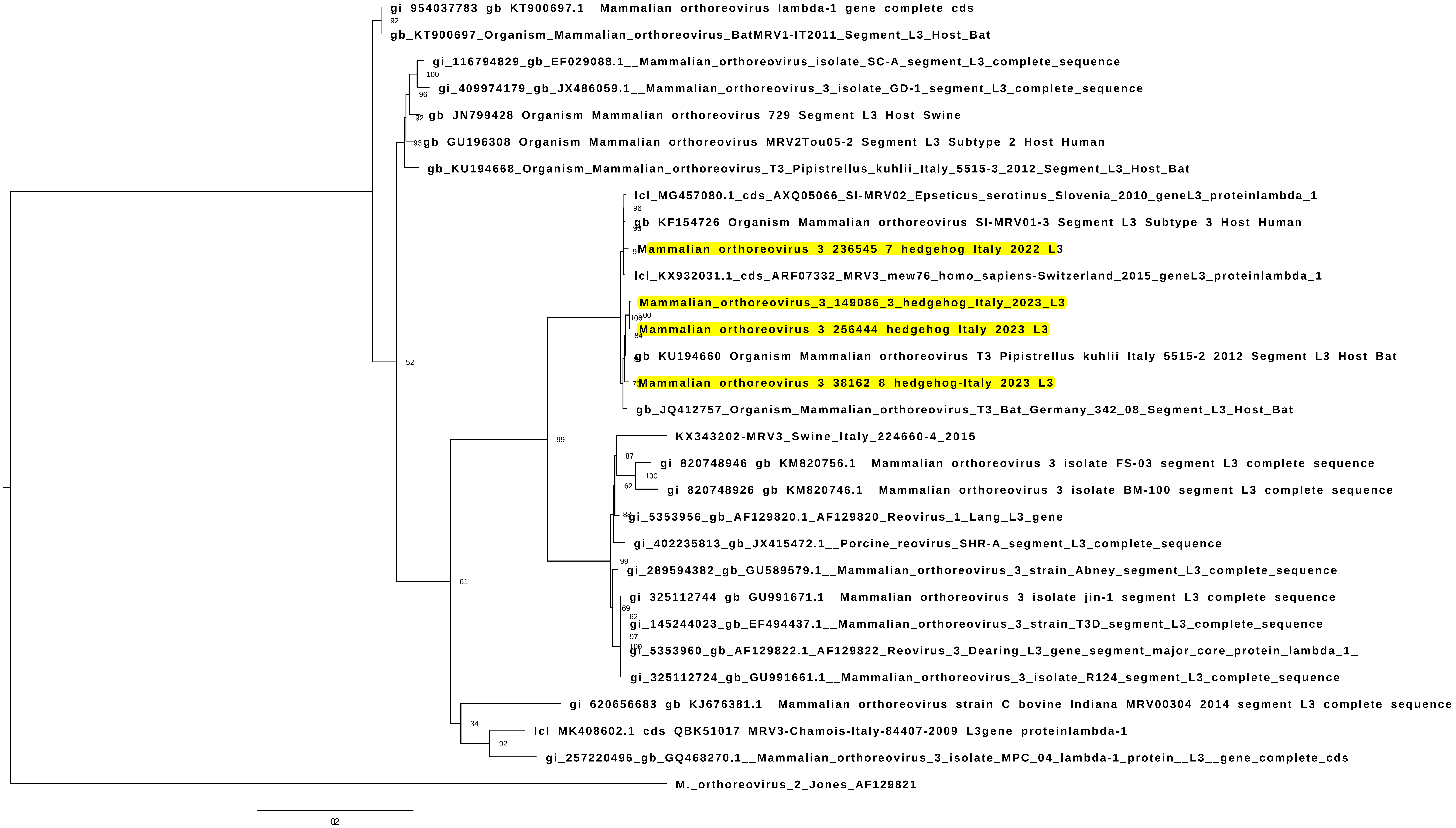

gi_954037783_gb_KT900697.1__Mammalian_orthoreovirus_lambda-1_gene_complete_cds
gb_KT900697_Organism_Mammalian_orthoreovirus_BatMRV1-IT2011_Segment_L3_Host_Bat
gi_116794829_gb_EF029088.1__Mammalian_orthoreovirus_isolate_SC-A_segment_L3_complete_sequence
gi_409974179_gb_JX486059.1__Mammalian_orthoreovirus_3_isolate_GD-1_segment_L3_complete_sequence
gb_JN799428_Organism_Mammalian_orthoreovirus_729_Segment_L3_Host_Swine
gb_GU196308_Organism_Mammalian_orthoreovirus_MRV2Tou05-2_Segment_L3_Subtype_2_Host_Human
gb_KU194668_Organism_Mammalian_orthoreovirus_T3_Pipistrellus_kuhlii_Italy_5515-3_2012_Segment_L3_Host_Bat
lcl_MG457080.1_cds_AXQ05066_SI-MRV02_Epseticus_serotinus_Slovenia_2010_geneL3_proteinlambda_1
gb_KF154726_Organism_Mammalian_orthoreovirus_SI-MRV01-3_Segment_L3_Subtype_3_Host_Human
Mammalian_orthoreovirus_3_236545_7_hedgehog_Italy_2022_L3
lcl_KX932031.1_cds_ARF07332_MRV3_mew76_homo_sapiens-Switzerland_2015_geneL3_proteinlambda_1
Mammalian_orthoreovirus_3_149086_3_hedgehog_Italy_2023_L3
Mammalian_orthoreovirus_3_256444_hedgehog_Italy_2023_L3
gb_KU194660_Organism_Mammalian_orthoreovirus_T3_Pipistrellus_kuhlii_Italy_5515-2_2012_Segment_L3_Host_Bat
Mammalian_orthoreovirus_3_38162_8_hedgehog-Italy_2023_L3
gb_JQ412757_Organism_Mammalian_orthoreovirus_T3_Bat_Germany_342_08_Segment_L3_Host_Bat
KX343202-MRV3_Swine_Italy_224660-4_2015
gi_820748946_gb_KM820756.1__Mammalian_orthoreovirus_3_isolate_FS-03_segment_L3_complete_sequence
gi_820748926_gb_KM820746.1__Mammalian_orthoreovirus_3_isolate_BM-100_segment_L3_complete_sequence
gi_5353956_gb_AF129820.1_AF129820_Reovirus_1_Lang_L3_gene
gi_402235813_gb_JX415472.1__Porcine_reovirus_SHR-A_segment_L3_complete_sequence
gi_289594382_gb_GU589579.1__Mammalian_orthoreovirus_3_strain_Abney_segment_L3_complete_sequence
gi_325112744_gb_GU991671.1__Mammalian_orthoreovirus_3_isolate_jin-1_segment_L3_complete_sequence
gi_145244023_gb_EF494437.1__Mammalian_orthoreovirus_3_strain_T3D_segment_L3_complete_sequence
gi_5353960_gb_AF129822.1_AF129822_Reovirus_3_Dearing_L3_gene_segment_major_core_protein_lambda_1_
gi_325112724_gb_GU991661.1__Mammalian_orthoreovirus_3_isolate_R124_segment_L3_complete_sequence
gi_620656683_gb_KJ676381.1__Mammalian_orthoreovirus_strain_C_bovine_Indiana_MRV00304_2014_segment_L3_complete_sequence
lcl_MK408602.1_cds_QBK51017_MRV3-Chamois-Italy-84407-2009_L3gene_proteinlambda-1
gi_257220496_gb_GQ468270.1__Mammalian_orthoreovirus_3_isolate_MPC_04_lambda-1_protein__L3__gene_complete_cds
M._orthoreovirus_2_Jones_AF129821
0.2
